# Therapeutic Effect of IL1β Priming Tonsil Derived-Mesenchymal Stem Cells in Osteoporosis

**DOI:** 10.1007/s13770-021-00350-3

**Published:** 2021-06-11

**Authors:** Minjoo Yoo, Sungkuk Cho, Sunhye Shin, Jung-Mi Kim, Hyeon-Gyeong Park, Sungyoo Cho, Yu Kyeong Hwang, Dae Hwi Park

**Affiliations:** Cell Therapy Research Center, Green Cross LabCell, 107, Ihyeon-ro 30 beon-gil, Giheung-gu, Yongin-si, Gyeonggi-do 16924 South Korea

**Keywords:** Palatine tonsil, Mesenchymal stem cells, Interleukin 1β, Osteoporosis

## Abstract

**Background:**

Stem cell therapies can be a new therapeutic strategy that may rebalance anabolic and anti-resorptive effects in osteoporosis patients. Tonsil-derived mesenchymal stem cells (TMSCs) can be an alternative therapeutic source for chronic degenerative diseases including osteoporosis. MSCs acquire immune regulatory function under the inflammatory cytokines. Since interleukin (IL) 1β is known to be one of inflammatory cytokines involved in osteoporosis progression, treatment of IL1β with TMSCs may enhance immunomodulatory function and therapeutic effects of TMSCs in osteoporosis.

**Methods:**

For IL1β priming, TMSCs were cultured in the presence of the medium containing IL1β for 1 day. Characteristics of IL1β priming TMSCs such as multipotent differentiation properties, anti-inflammatory potential, and suppression of osteoclast differentiation were assessed *in vitro*. For *in vivo* efficacy study, IL1β priming TMSCs were intravenously infused twice with ovariectomized (OVX) osteoporosis mouse model, and blood serum and bone parameters from micro computed tomography images were analyzed.

**Results:**

IL1β priming TMSCs had an enhanced osteogenic differentiation and secreted factors that regulate both osteoclastogenesis and osteoblastogenesis. IL1β priming TMSCs also suppressed proliferation of peripheral blood mononuclear cells (PBMCs) and decreased expression of Receptor activator of nuclear factor kappa-Β ligand (RANKL) in PHA-stimulated PBMCs. Furthermore, osteoclast specific genes such as Nuclear factor of activated T cells c1 (NFATc1) were effectively down regulated when co-cultured with IL1β priming TMSCs in RANKL induced osteoclasts. In OVX mice, IL1β priming TMSCs induced low level of serum RANKL/osteoprotegerin (OPG) ratio on the first day of the last administration. Four weeks after the last administration, bone mineral density and serum Gla-osteocalcin were increased in IL1β priming TMSC-treated OVX mice. Furthermore, bone formation and bone resorption markers that had been decreased in OVX mice with low calcium diet were recovered by infusion of IL1β priming TMSCs.

**Conclusion:**

IL1β priming can endow constant therapeutic efficacy with TMSCs, which may contribute to improve bone density and maintain bone homeostasis in postmenopausal osteoporosis. Therefore, IL1β priming TMSCs can be a new therapeutic option for treating postmenopausal osteoporosis.

**Supplementary Information:**

The online version contains supplementary material available at 10.1007/s13770-021-00350-3.

## Introduction

Osteoporosis is a systemic bone disease characterized by reduced bone density and deterioration of micro-architecture of bone tissue. Prevalence of osteoporosis is higher in elderly population. In particular, one in three women over age 50 has higher hip fracture and mortality rates due to postmenopausal osteoporosis [1]. Although anti-catabolic drugs such as bisphosphonate and anabolic drugs, PTH and anti-sclerostin, are available to treat postmenopausal osteoporosis, these drugs may be used for a limited period of time in some patients due to high risk of side effects [2–5]. Furthermore, most of the drugs used for osteoporosis cannot restore bone loss in patients. Therefore, there are still clinical unmet needs for developing new drugs including stem cell therapy that not only prevents the bone loss, but also induces recovery from the bone loss by rebalancing bone homeostasis with minimum side effects [6].

Mesenchymal stem cells (MSCs) can be isolated from various tissues such as adipose tissue, bone marrow, and umbilical cord blood. MSCs have multipotent differentiation property into bone, cartilage, fat, and others. MSCs based therapy may be a good candidate for the new therapeutic options that aim for both anabolic and anti-resorptive effects on bones in osteoporosis. Bone marrow derived mesenchymal stem cells (BM-MSCs) have been used in the most stem cell based studies for osteoporosis. Since isolation of the bone marrow from a donor is an invasive procedure and yield of BM-MSC isolation from the bone marrow may not be sufficient for commercial scale manufacturing, application of MSCs from alternative sources has been investigated for osteoporosis treatment.

Accumulating evidence suggests that human tonsil derived mesenchymal stem cells (TMSCs) may have potential to regenerate damaged tissues in inflammatory disease [7, 8], endocrine disease [9, 10], and osteoporosis [11–13]. TMSCs constitutively expressed anti-inflammatory factors such as PD-L1 and interleukin-1 receptor antagonist (IL-1RA) [14]. Furthermore, conditioned medium from TMSCs attenuated Th17 mediated inflammation in psoriatic mouse model [15]. In addition, TMSCs secreted osteoprotegerin (OPG) that is able to regulate Th17-induced osteoclastogenesis [16]. Based on these finding, TMSCs is likely to be an ideal source of stem cell therapy for osteoporosis, which have both anti- inflammatory function and ability to recover bone balance.

Postmenopausal osteoporosis results from lack of estrogen that may lead to an increase in pro-inflammatory cytokines and imbalance between bone resorption and bone formation [17, 18]. In pathological condition in osteoporosis, pro-inflammatory cytokines such as tumor necrosis factor alpha (TNFα), IL1β, and IL17 were increased [19–21]. A previous study demonstrated that IL1β induced an increase of RUNX2 expression in bone marrow stem cells and promoted differentiation into osteoblasts via Wnt5a pathway [22]. However, biological effects of systemically infused IL1β priming TMSCs in postmenopausal osteoporosis model have not been investigated. In this study, we first investigated whether IL1β priming TMSCs modulate immune cell function and osteoclast differentiation *in vitro*. Subsequently, we also examined effects of intravenous infusion of IL1β priming TMSCs on recovery from bone loss in ovariectomized osteoporosis mouse model.

## Methods

### Animal model for osteoporosis and systemic infusion of IL1β priming TMSCs

6 weeks old female Balb/C mice were purchased from Orient Bio Inc. (Seongnam, Korea). All experiments were performed in accordance with guideline of Institutional Animal Care and Use Committee of GC pharma animal research center (Yong-in, Korea). Osteoporosis was induced in 7 weeks old Balb/C mice by performing ovariectomy (OVX) under general anesthesia using isoflurane (JW pharma, Seoul, Korea). For sham group mice, same surgical procedure was performed, but ovaries were not removed. After surgery, sham and OVX groups were fed low-calcium diet (0.01% low calcium diet, TD.95027, Harlan Laboratories, Inc., Indianapolis, IN, USA). After 8 weeks, mice were intravenously injected once a month for 2 months with vesicle (0.1 mL PBS) or TMSCs (8 × 10^5^ cells in 0.1 mL PBS). Bones were dissected and fixed in 10% neutral formalin solution (Sigma-Aldrich, St. Louis, MO, USA) overnight for histological analysis and micro-CT analysis.

### Culture of mesenchymal stem cells

Tonsil tissue was provided by Prof. Han Su Kim and Prof. Soo Yeon Jung (IRB approved by Ewha Womans University Mok-Dong hospital, Seoul, Korea; no. ECT 11-53-02). Isolation of TMSCs were performed using a method described previously with modification [23]. Adherent primary TMSCs were maintained in high glucose DMEM (Gibco, Grand Island, NY, USA) containing 10% fetal bovine serum with 1X antibiotic-antimycotic (Gibco). TMSCs from passages 6 through 7 were used for this study. For IL1β priming, TMSCs were cultured in the presence of IL1β (10 ng/mL, Peprotech) containing medium for 24 h. For co-culture with osteoclast, TMSCs were seeded onto the upper 0.4 μm polyethylene terephthalate membrane chamber of transwell plate (Corning, Corning, NY, USA). For IL1β priming, TMSCs in transwell were changed with IL1β (10 ng/mL) containing medium. After 24 h, IL1β priming TMSCs were washed twice with PBS and used for co culture with osteoclast in the lower chamber.

BM-MSCs were purchased from Lonza and cultured in the same medium. BM-MSCs from passages 4 through 5 were used for this study and IL1β priming of BM-MSCs were performed in the same way as described above.

### Multi-lineage differentiation assays of TMSCs

To induce osteogenic or adipogenic differentiation, confluent TMSCs were incubated with osteogenesis or adipogenesis differentiation media respectively. All the reagents and differentiation kits in this study were purchased from Lonza. Calcium deposition was evaluated by Alizarin Red S staining after 3 weeks of osteogenic differentiation. Oil Red O staining was used to assess lipid droplet after 3 weeks of adipogenic differentiation. To induce chondrogenic differentiation, spherical cell pellets were cultured in chondrogenesis differentiation media for 6 weeks. Sections were counterstained with nuclear fast red and evaluated cartilage matrix deposition by Alcian blue staining. Samples were examined using light microscopy (Olympus, Tokyo, Japan).

### Osteoclast induction of RAW 264.7 and co-culture with TMSCs

Murine monocytic cell line RAW 264.7 (ATCC, Manassas, MA, USA) was maintained in the same medium used in TMSC culture. Osteoclastogenesis of monocytes/macrophages was conducted using a method previously described with minor modification [24]. For induction into osteoclast, RAW 264.7 were cultured in TMSC culture medium with RANKL (100 mg/mL, Peprotech, Cranbury, NJ, USA) and PD98059 (20uM, Gibco). After 2 days, RAW 264.7-derived osteoclastic cells were co-cultured with TMSCs. After 1 days, osteoclast related genes were evaluated by Quantitative PCR and number of NFATc1 expressing cells was analyzed by flow cytometry analysis.

### Flow cytometry

TMSCs were characterized using several cell surface markers by flow cytometry. Cell surface staining was performed with fluorescent conjugated antibodies for 30 min at 4 °C in the dark chamber. All antibodies used for surface staining were purchased from eBioscience and BD Pharmigene. RAW 264.7-derived osteoclasts were fixed and permeabilized using BD Cytofix/Cytoperm Solution Kit (BD Biosciences, Bedford, MA, USA) and stained with PE-conjugated NFATc1 antibody (Biolegned). All sample were analyzed with FACS-LSR Fortessa (BD Bioscience) and data were analyzed with FlowJo software.

### CFSE proliferation assay

Human peripheral blood mononuclear cells (PBMC) were purchased from ALLCELL Technologies. PBMCs were labelled with 3 μM carboxyfluorescein succinimidyl ester (CFSE, Thermo Fisher) for 4 min at room temperature and was washed twice with culture medium. Co-culture was established by incubating 6 × 10^5^ PBMCs and 3 × 10^4^ TMSCs per well in 24-well plates with culture medium (RPMI-1640 medium supplemented with 10% fetel bovine serum, 1% antibiotic antimycotic) for 6 days. PHA-M was added to stimulate T cell proliferation. Flow cytometry analysis for cell division by dilution of CFSE was conducted to assess PBMC proliferation.

### Real-time PCR

Total RNA was extracted using RNeasy plus kit (Qiagen,, Valencia, CA, USA) and was transcribed to cDNA using Maxima First Strand cDNA Synthesis Kit (Thermo Fisher Scientific, Waltham, MA, USA). Quantitative PCR was performed using QuantStudio™ 5 Real-Time PCR System (Applied Biosystems) with TB Green® Premix Ex Taq™ II master mix (Takara, Kyoto, Japan). Gene specific primers sequences are listed in supplement Table S1.

### Enzyme-linked immunosorbent assay (ELISA)

TMSCs conditioned medium was analyzed by ELISA according to the manufacturer’s instructions for human OPG (Thermo Fisher Scientific) and human IL6 (R&D system, Minneapolis, MN, USA). Blood was collected from venous cava under general anesthesia and clotted at room temperature for 2 h. Serum was preserved at − 80 °C until analysis. Serum analysis was performed according to the manufacturer’s instructions for mouse RANKL (R&D systems), mouse OPG (R&D systems), mouse Gla-osteocalcin (Takara), and mouse CTX-1 (LSBio, Seattle, WA, USA).

### Western blot analysis

TMSCs were washed twice with PBS and lysed in RIPA lysis buffer supplemented with cocktail of protease/phosphatase inhibitors (Thermo Fisher Scientific). Supernatants were collected after centrifugation at 12,000 rpm for 10 min. Humerus was frozen rapidly using liquid nitrogen immediately after dissection. Frozen humerus was homogenized using a mortar and pestle on dry ice. Whole protein of bone was extracted in ice by RIPA lysis buffer supplemented with cocktail of protease/phosphatase inhibitors for 20 min. Supernatants were collected after centrifugation at 12,000 rpm for 10 min and protein concentration was measured using BCA protein assay kit (Pierce). Primary antibodies used were anti-Wnt16 (Thermo Fisher Scientific), anti-RUNX2, anti-osteocalcin, anti-CathepsinK (Santa Cruz Biotechnology Inc., Dallas, TX, USA), and anti-beta actin (Sigma-Aldrich) antibodies. Secondary antibodies used were HRP conjugated antibodies. Protein bands were visualized with ECL™ Prime Western Blotting Detection Reagent (Amersham) in GBOX chemi XX9 (Syngene).

### Histological analysis

For histological analysis, isolated femurs from euthanatized mice were fixed with 0.1 M phosphate buffer containing 4% paraformaldehyde (Sigma-Aldrich) at 4 °C for a 3 days. Then they were decalcified in 14.5% EDTA solution (sigma) for 1 weeks at room temperature. Decalcified tissues were rinsed, dehydrated, and paraffin embedded in accordance with general protocols. Paraffinized specimens were sectioned in 4 μm thickness with a microtome. Histological study was performed with Harris hematoxylin (BBC Biochemical, Vernon, WA, USA) and Eosin Y (BBC Biochemical). Images were obtained by inverted Nikon TS2R-FL microscope (Nikon).

### Micro CT measurement

Evaluation of trabecular structure in distal femoral bones was performed using micro-computed tomography (micro-CT) (Skyscan 1072, Kontich, Belgium). Fixed distal femurs in 4% PFA were scanned at 14.85 μm pixel size with 50 kVp, 200 μA, 0.5 mm AI filter. Data were reconstructed using NRecon software and Tomographic image were analyzed by CTscan software (ver 1.6).

### Statistical analysis

Data from quantitative experiments are represented as means standard error of the mean. One way Anova or T-test with Bonferroni Correction was calculated using Graphpad prism 5 software. For all tests, a significance level of 5% was assigned.

## Results

### IL1β priming TMSC expresses bone homeostasis-related components

BM-MSCs-treated with IL1β at low concentration (0.1 ng/mL) had enhanced differentiation potential into osteoblast [22]. Nevertheless, it is not well understood whether pre- or continuous treatment of IL1β increase differentiation potential of TMSCs to osteoblast. Therefore, we examined whether pre-treatment with IL1β for 1 day (IL1β priming) before induction of osteogenic differentiation enhances osteogenic potential of TMSCs.

**Fig. 1:**
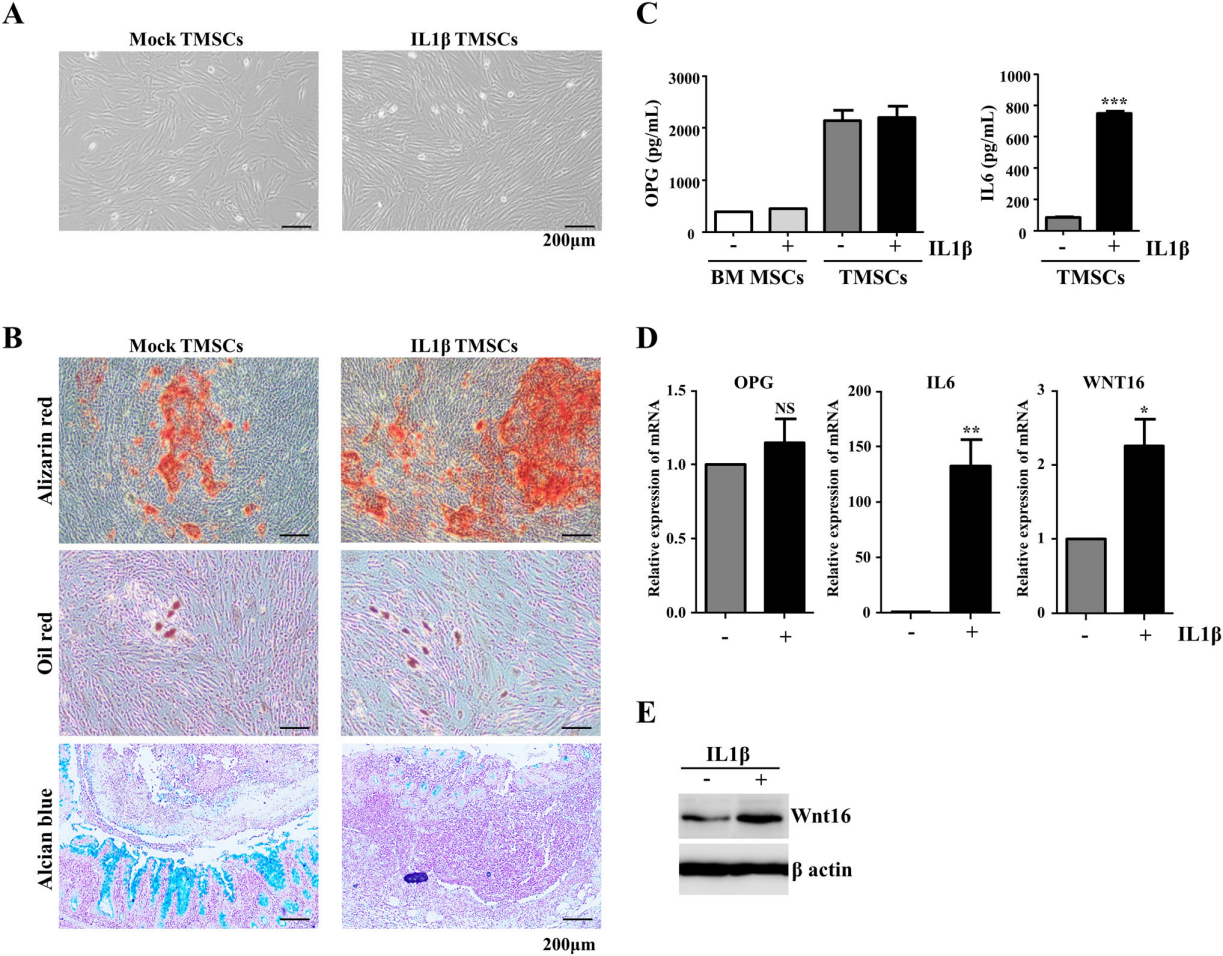
Characterization of IL1β priming TMSCs and their bone related properties. **A** Cell morphology, **B** Multi-potency of Mock and IL1β priming TMSCs. scale bar 200 µm, **C** ELISA analysis of OPG and IL6 in BM MSC and TMSC conditioned medium at day 1. **D**. Quantitative PCR analysis of mRNA expression level of OPG, IL6, and WNT16 in TMSCs. **E**. Western blot analysis of Wnt16 expression in mock and IL1β priming TMSC lysates. *p < 0.05 **p < 0.01 ***p < 0.001 compared with Mock TMSCs

TMSCs-treated with IL1β for 1 day had no difference in its morphology and MSC surface CD marker expression profile compared to control (Supplementary Fig. 1, Fig. 1A). However, our preliminary RNAseq analysis showed that pro-osteogenic transcription factors and signaling factors such as RUNX2 (1.48 fold), TWIST 2 (1.79 fold), IGF2 (3.5 fold), BMP2 (12.66 fold), BMP7 (6.55 fold), WNT10A (2.03 fold), and WNT16 (2.88 fold) were differentially increased in IL1β priming TMSCs compared to mock control (Data not shown). In comparison with mock TMSCs, IL1β priming TMSCs produced more alizarin red S stained osteoblasts after osteogenic induction for 21 days but did not efficiently produce Alcian blue stained chondrocytes when inducing chondrogenic differentiation (Fig. 1B). However, there was no difference in adipogenic differentiation potential between mock and IL1β priming TMSCs (Fig. 1).

OPG secreted by osteoblasts in bone was able to prohibit excessive bone resorption [25, 26]. IL6 is an immunomodulatory cytokine that can be induced by IL1β signaling in MSC [27]. As predicted, high expression and secretion of IL6 but no significant increase in the secretion of OPG was observed in IL1β priming TMSCs compared to mock TMSCs (Fig. 1C, D). Interestingly, expression of Wnt16 was significantly increased in IL1β priming TMSCs (Fig. 1D). These results suggest that IL1β priming TMSCs have enhanced osteogenic differentiation potential and secrete components such as IL6 and Wnt16 that are able to regulate osteoclast and osteoblast differentiation in the bone.

### IL1β priming TMSCs decrease osteoclast related gene expression in RANKL-induced differentiation

We confirmed that IL6 decreased expression of osteoclast genes such as c-fos and NFATc1 under the RANKL concentration of 100 ng/ml in RAW 264.7-derived osteoclasts (Supplementary Fig. 2). However, expression of cathepsin K (ctsk) was decreased only in the presence of OPG (50 ng/mL). Interestingly, treatment of both IL6 and OPG synergistically suppressed expression of osteoclast genes (Supplementary Fig. 2). As shown in Fig. 1C, D, IL1β priming TMSCs was able to secrete OPG and IL6. Therefore, we hypothesized that IL1β priming TMSCs may also suppress osteoclast differentiation by secretion of OPG and IL6.

**Fig. 2:**
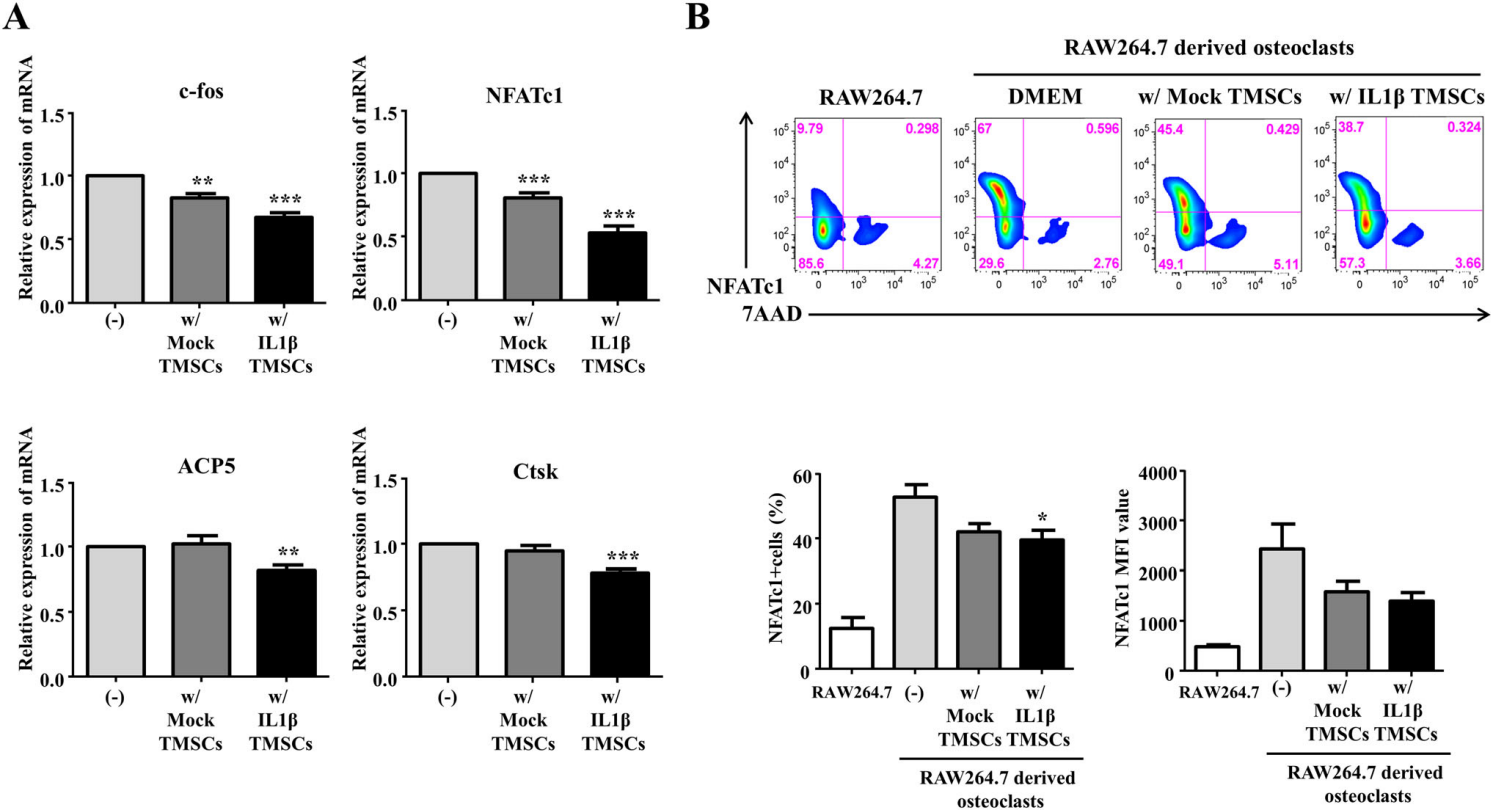
Effects of IL1β priming TMSCs on RAW 264.7 cell-derived osteoclast differentiation. **A** Quantitative PCR analysis of mRNA expression levels of the osteoclast markers in osteoclasts co-cultured with Mock or IL1β priming TMSCs. **B** Representative FACS images of NFATc1 expression in osteoclasts co-cultured with Mock or IL1β priming TMSCs

To see whether IL1β priming TMSCs suppress osteoclast differentiation, IL1β priming TMSCs were co-cultured with RAW 264.7-derived osteoclasts. IL1β priming TMSC significantly decreased the RAW 264.7 cell population that strongly expressed NFATc1 (Fig. 2). Furthermore, IL1β priming TMSCs not only suppressed expression of osteoclast commitment genes such as c-fos and NFATc1, but also resorption enzyme genes such as ACP5 and cathepsin K in RAW 264.7-derived osteoclasts. However, mock TMSCs suppressed expression of early differentiation genes for osteoclast commitment, but did not affected expression of resorption enzymes that are related with osteoclast activity. These results showed IL1β priming TMSCs have suppressive effect on differentiation of RANKL-induced osteoclasts.

### IL1β priming TMSCs suppress PBMC proliferation and decrease RANKL expression in PHA-stimulated PBMCs

**Fig. 3:**
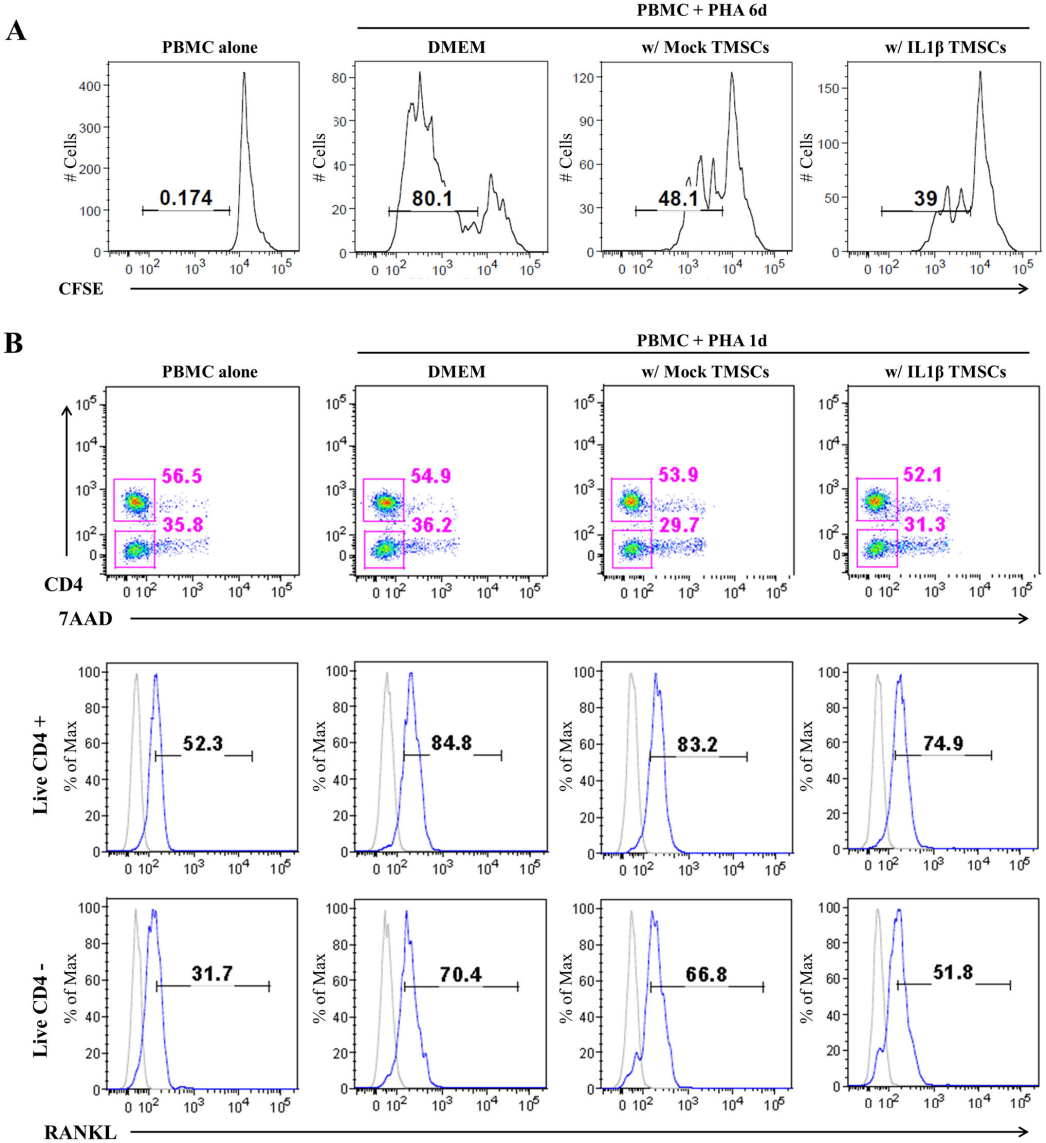
Immunomodulatory effects of IL1β priming TMSCs on PHA-stimulated PBMCs. **A** Representative FACS images of proliferation of PBMC co-cultured with Mock or IL1β priming TMSCs. **B** Representative FACS images of RANKL expression on PHA-stimulated PBMCs co-cultured with Mock or IL1β priming TMSCs

To investigate immunomodulatory function of mock and IL1β priming TMSC, TMSCs were co-cultured with PBMCs that had been labeled with CFSE to track cell division. 6 days after PHA stimulation (Day 6), CFSE low PBMC population was decreased about 10% when they were co-cultured with IL1β priming TMSCs compared to that were co-cultured with mock TMSCs (Fig. 3A).

In addition, at Day 1, RANKL expression in all the CD4 positive and CD4 negative cells was decreased about 8.3% and 15%, respectively when co-cultured with IL1β priming TMSCs. However, mock TMSC did not affect RANKL expression in PHA-stimulated PBMCs. (Fig. 3B). These results suggested that IL1β priming TMSCs have immunomodulatory capacity to regulate immune cells in osteoporotic development.

### Treatment of IL1β priming TMSCs increases bone mineral density in OVX mice

**Fig. 4:**
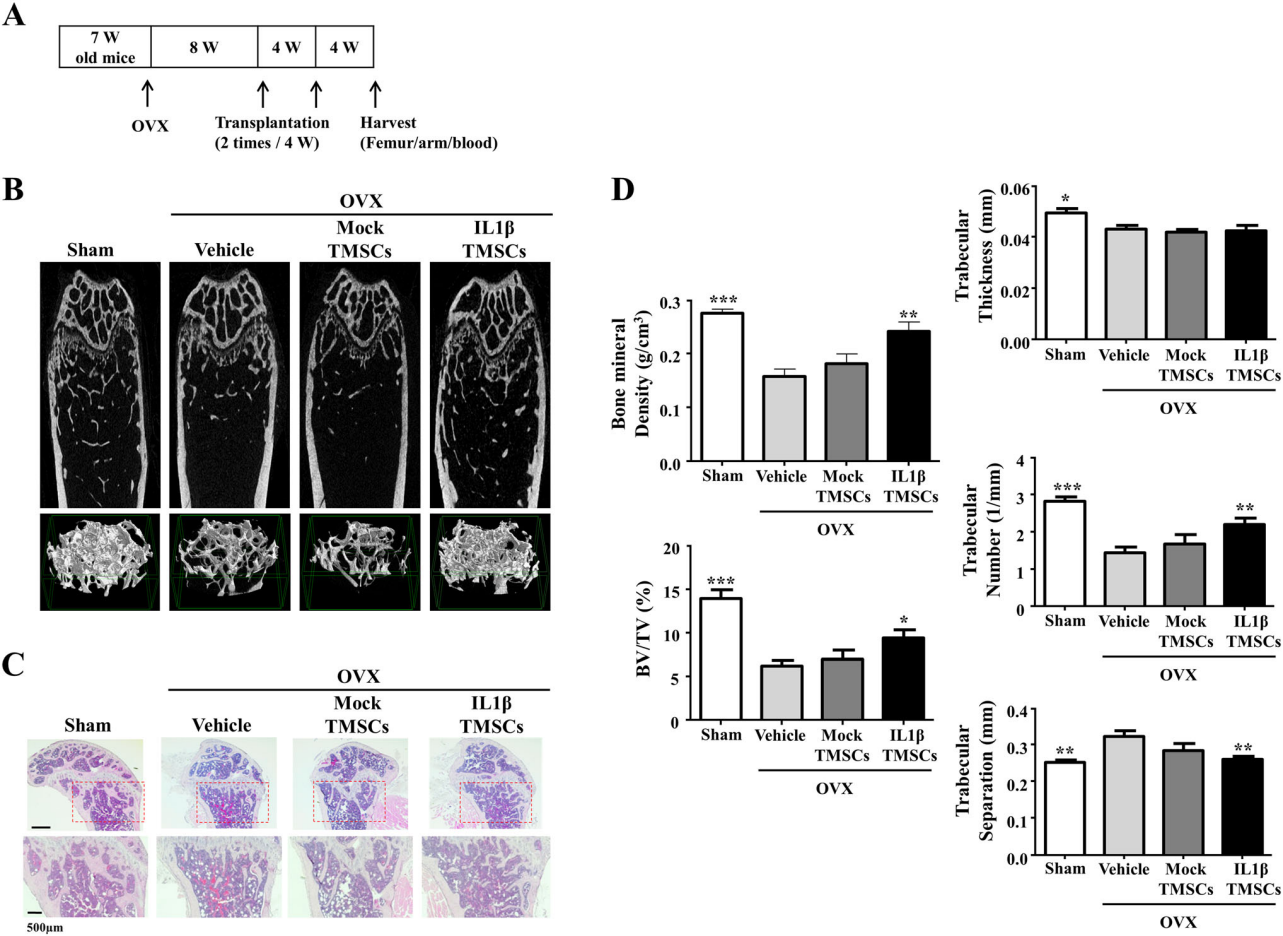
Therapeutic effects of systemic infusion of IL1β priming TMSCs in ovariectomized osteoporosis mouse model. **A** Experiment scheme. **B** Representative micro CT image of the femurs harvested at 8 weeks after cells injection. **C** Representative H&E stained sections of femurs. **D** Quantitative analysis of trabecular bone parameters, including bone volume/tissue volume, bone mineral density, trabecular thickness, trabecular numbers, and trabecular separation. *p < 0.05 **p < 0.01 ***p < 0.001 compared with OVX-vehicle

To study whether IL1β priming TMSCs have therapeutic potential in osteoporosis, we established an animal model for osteoporosis by surgical removal of ovaries in 7 weeks old female mice followed by low calcium diet to enhance bone loss (OVX model). 8 weeks after ovariectomy, we confirmed osteoporotic features in the bone of OVX mice such as significant loss of trabecular bone volume due to significant decrease of trabecular thickness, BV/TV, and trabecular number (supplementary Fig. 3). At this point, IL1β priming TMSCs or mock TMSCs were intravenously injected into the OVX mice (twice, 4 weeks apart). Four weeks after the last administration, all mice were sacrificed, and bone tissues were examined. (Fig. 4A).

As shown in representative micro CT images in Fig. 4B, structural impairment of trabecular microarchitecture in control OVX mice was improved in IL1β priming TMSC-treated OVX mice compared with mock TMSC-treated OVX or vehicle-treated OVX mice. Corresponding to the micro CT image analysis, histological examination of H&E staining sections from proximal femurs also showed that number of trabecular in IL1β priming TMSC-treated OVX mice was increased compared to vehicle or mock TMSC-treated OVX mice (Fig. 4C).

In quantitative analysis of micro CT images, Trabecular BMD (*p* = 0.004), BV/TV (*p* = 0.02), and trabecular numbers (*p* = 0.008) were significantly increased, but trabecular separation (*P* = 0.006) was significantly decreased in IL1β priming TMSC-treated OVX mice compared with vehicle-treated OVX mice (Fig. 4D). However, trabecular thickness was decreased in all the OVX mice compared with normal mice, and was not recovered in IL1β priming TMSC-treated OVX group. Overall, treatment of mock TMSC did not show significant changes compared to vehicle-treated OVX mice. (Fig. 4D). These results suggested that IL1β priming TMSCs have therapeutic potential to improve estrogen deficiency induced osteoporosis.

### Treatment of IL1β priming TMSCs restores both bone formation and resorption markers in OVX mice

**Fig. 5:**
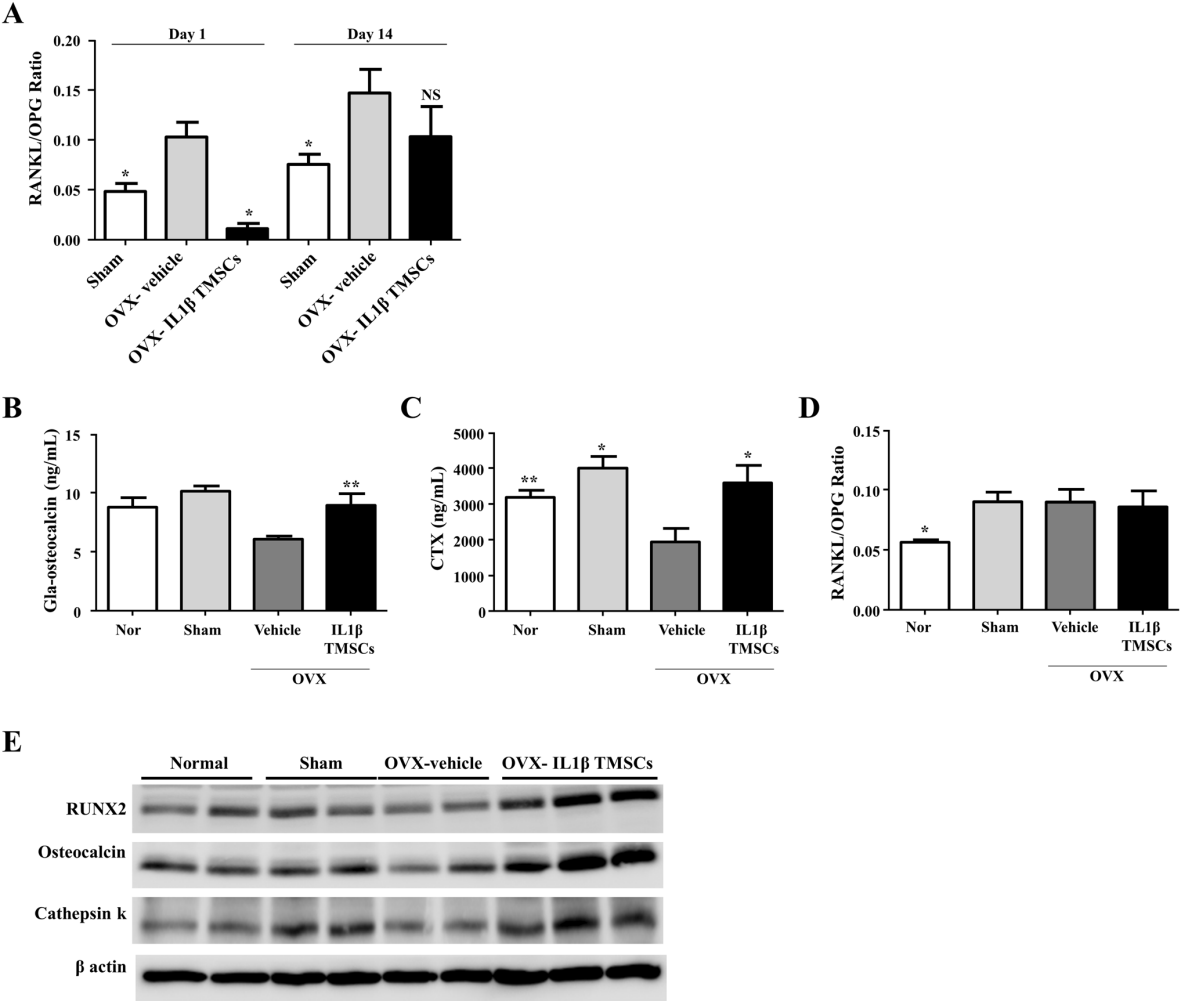
Function of IL1β priming TMSCs for balance between bone formation and resorption markers. **A** RANKL/OPG ratio in serum harvested at day 1 and day 14 after IL1 β priming TMSC infusion via ELISA assay (n = 3). *p < 0.05 compared with OVX-vehicle. **B**–**D** Quantification of Gla-osteocalcin and CTX and RNAKL/OPG ratio in serum harvested 8 weeks after IL1β priming TMSC injection (n = 5 or 6) using ELISA assay. *p < 0.05 **p < 0.01 compared with OVX-vehicle. **E** Western Blot analysis of whole humerus lysates (n = 2 or 3)

To examine systemic effects of IL1β priming TMSCs in OVX mice, blood serum was analyzed. OVX mice had relatively high RANKL/OPG, an index of osteoclastogenic stimulation [28, 29], compared with that of normal mice. However, in IL1β priming TMSC-treated OVX mice, serum RANKL/OPG ratio was significantly decreased on the first day of administration compared to sham control or vehicle-treated OVX mice, but this difference was not significant on 14 days after intravenous administration of IL1β priming TMSCs (Fig. 5A, B). 4 weeks after second administration of vehicle or IL priming TMSCs, serum RANKL/OPG ratio was similar level in all OVX mice group. Thus, these results suggested that serum RANKL/OPG ratio was temporally regulated immediately after systemic infusion of IL1β priming TMSCs.

16 weeks after ovariectomy, both bone formation marker, Gla-osteocalcin, and bone resorption marker, CTX-1, were reduced in vehicle-treated OVX mice compared to that of normal or sham mice. Serum Gla-osteocalcin in sham mice which were fed calcium diet was also high compared to that of normal mice. However, administration of IL1β priming TMSCs restored not only serum Gla-osteocalcin level but also serum CTX-1 level in OVX mice (Fig. 5C, D).

Corresponding to the serum analyses, protein levels of both bone formation marker (RUNX2 and osteocalcin) and resorption maker (cathepsin K) were recovered in whole bone tissue of IL1β priming TMSC-treated OVX mice (Fig. 5E). Overall, these results suggested that IL1β priming TMSCs have potential to restore bone homeostasis in osteoporosis.

## Discussion

Stem cell therapy that can ensure the constant therapeutic efficacy to rebalance bone homeostasis are mandatory for the successful treatment of osteoporosis. However, administration of patient’s own MSCs or MSCs from alternative sources still exhibited a short-term engraftment and limited efficacy to rebalance bone formation and resorption in osteoporosis [24, 30]. Human palatine tonsils can serve as an alternative source for MSCs [23]. Human tonsil tissue can be obtained from tonsillectomy and average amount of adherent primary TMSCs obtained from 1 g of the tonsil tissue was about 1.3 × 10^7^ at passage 0. Number of the tonsil-derived MSCs (TMSCs) at passage 7 can reach up to 3.5 × 10^13^ (unpublished data). Thus, tonsils can be a good source that are able to provide commercial scale TMSCs for cell therapy by isolation of MSCs from a single donor.

Recent study showed that systemic infusion of naïve TMSCs or conditioned medium in senile osteoporosis model mice showed reduction of visceral adipose tissue mass, but bone loss was not significantly recovered in these mice [11]. Interestingly, intra-tibial injection of naïve TMSCs in ovariectomized postmenopausal osteoporosis model mice improved bone mineral density compared to untreated animals [12]. Moreover, subcutaneous injection of TMSCs-embedded hydrogel led to better recovery of the femoral heads in ovariectomized postmenopausal osteoporosis model mice [13]. These results suggest that local administration of TMSCs has therapeutic benefits for osteoporosis. However, therapeutic potential of systemic infusion of naïve or priming TMSCs for postmenopausal osteoporosis has not been demonstrated yet.

In this study, we investigated the therapeutic potential of systemic infusion of TMSCs primed by an inflammatory cytokine (IL-1β) that mimic inflammation increased by the loss of estrogens at menopause [19, 31]. TMSCs expressed typical MSC surface marker such as CD73, CD90 and CD105 and expression of the markers was not changed after IL1β priming. However, IL1β priming TMSCs suppressed proliferation of PHA-stimulated PBMCs better than mock TMSCs. In addition, RANKL expression in PHA-stimulated PBMCs was also efficiently suppressed by IL1β priming TMSCs compared to mock TMSCs. These results support that anti-inflammatory effects of TMSCs are enhanced by IL1β priming.

OPG is largely expressed by osteoblast lineage cells of bone and may prevent unnecessary bone resorpion by acting as a decoy receptor to inhibit RANK-RANKL binding in osteoclast differentiation [25, 26]. It has been shown that TMSCs expressed significantly higher levels of OPG compared to bone marrow or adipose derived mesenchymal stem cells [16]. Our results also support higher OPG secretion in TMSCs compared to BM-MSCs. IL6 is a multifunctional cytokine which coordinates not only immune responses but also bone homeostasis [32–34]. In fact, IL6 has dual functions either as a negative or positive effector on osteoclastogenesis [35, 36]. It is well known that IL6 expression can be enhanced by IL1β priming in MSCs. Although IL6 modulates RANKL expression in osteoblasts and indirectly promotes functional activity and maturation of RANKL-induced osteoclasts in the existence of soluble receptor (sIL-6R), many studies also showed that IL6 may directly inhibit RANKL-induced osteoclast differentiation [36–38].

Osteoclast differentiation genes such as c-fos, NFATc1, ACP5, and Ctsk that are related with RAW 264.7-derived osteoclast formation were suppressed by IL1β priming TMSCs compared to mock TMSC. In particular, NFATc1, a key master transcription factor of osteoclast differentiation, was downregulated to the protein level in undifferentiated RAW 264.7. Furthermore, expression of c-fos and NFATc1 was inversely proportional to the concentration of IL6, and co-treatment of OPG with IL6 enhanced the IL6 mediated inhibition of c-fos and NFACTc1 in our osteoclast assay. Thus, these results suggest that high levels of inherent OPG secretion and IL1β induced IL6 secretion in IL1β priming TMSCs may promote inhibition of osteoclast gene expression and RANKL expression of immune cells.

Interestingly, expression of Wnt16, a positive regulator of cortical and trabecular bone mass and structure [39, 40], was also increased in IL1 priming TMSCs. Previous studies demonstrated that Wnt16 increased OPG expression in osteoblasts and regulated osteoclast formation in conjunction with Wnt5a [40, 41]. In addition, overexpression of Wnt16 increased mainly trabecular bone mass *in vivo* [40, 42]. For this reason, we hypothesized that IL1β priming TMSCs have constant and enhanced therapeutic efficacy in osteoporosis.

In this study, IL1β priming TMSCs were systemically infused in ovariectomized postmenopausal osteoporosis (OVX) model mice established by combining full bilateral ovariectomy with low calcium diet to insure bone mineral loss. Two months after systemic cell infusion, IL1β priming TMSCs restored BMD to normal mice level, but mock TMSCs did not have significant effects on BMD in OVX mice. Six-weeks low calcium diet significantly decreased bone resorption markers such as urinary excretion of deoxypyridinoline and serum tartrate-resistance acid phosphatase [43, 44]. Interestingly, we also found that serum RANKL OPG ratio was significantly decreased in OVX mice after IL1β priming TMSC infusion. Serum CTX-1, an index of bone resorption, was decreased in OVX mice with low calcium diet. Infusion of IL1β priming TMSCs restored both serum Gla-osteocalcin, an index of bone formation, and serum CTX-1 in OVX mice compared to vehicle control. In addition, expression of RUNX2, osteocalcin, and cathepsin K were also increased in whole bone of IL1β priming TMSC injected OVX mice compared to vehicle control. These results suggest that IL1β priming TMSCs may lead to modulate inflammation and recover both bone formation and resorption in postmenopausal osteoporosis, which may contribute to bone homeostasis.

In conclusion, we suggested that IL1β priming TMSCs has therapeutic potential for postmenopausal osteoporosis, which may improve bone density, restore bone homeostasis, and enhance anti-inflammatory capacity.

## Supplementary Information

Below is the link to the electronic supplementary material.

Supplementary file1 (TIF 1784 kb)

Supplementary file2 (TIF 942 kb)

Supplementary file3 (TIF 1992 kb)
