# Supplementary figures and images for: Therapeutic Effect of IL1β Priming Tonsil Derived-Mesenchymal Stem Cells in Osteoporosis
Source: Tissue Eng Regen Med. 2021 Jun 11;18(5):851–62. doi: 10.1007/s13770-021-00350-3 (PMC8440756; doi:10.1007/s13770-021-00350-3)

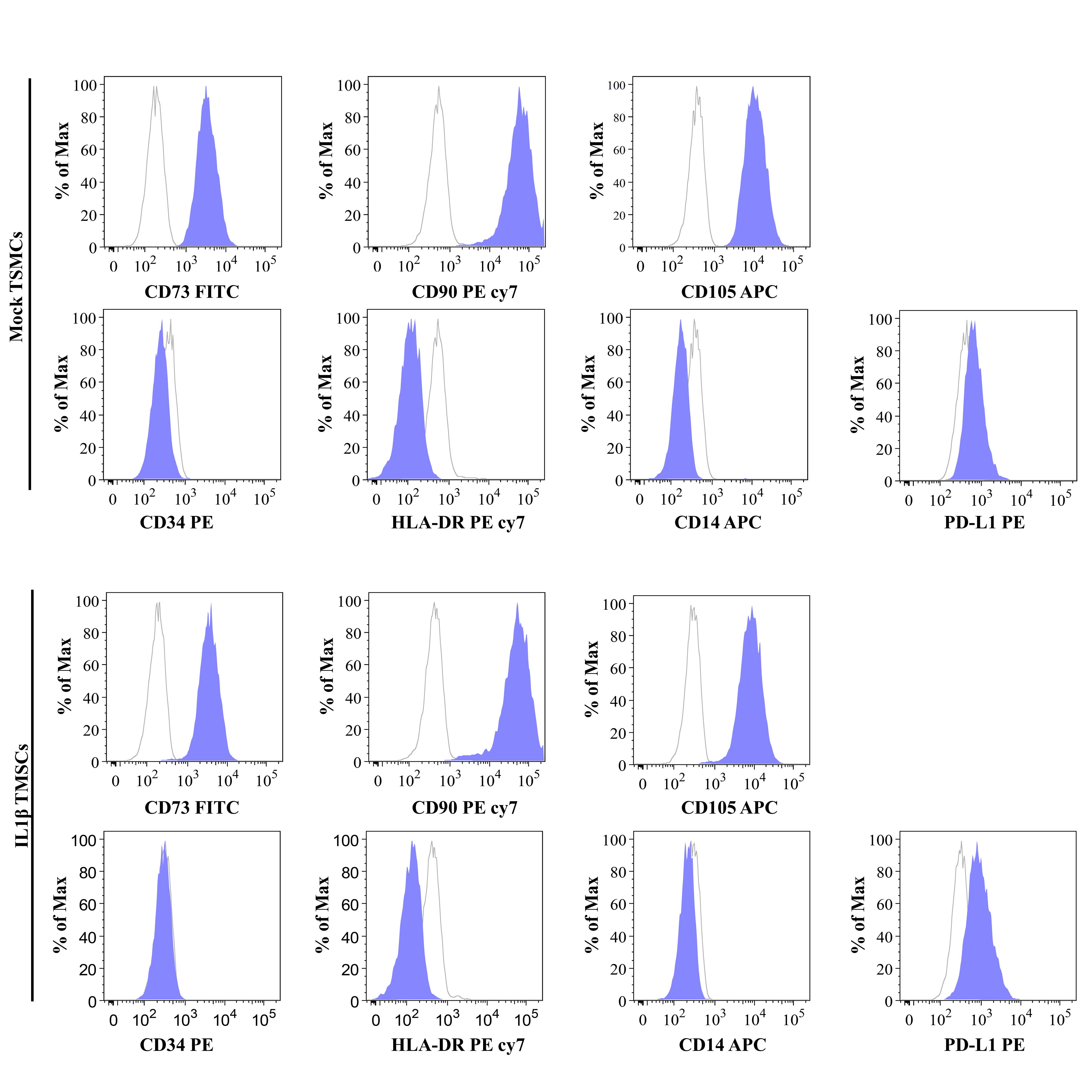

Supplement: Supplementary file 1 — Supplementary file1 (TIF 1784 kb) [file 13770_2021_350_MOESM1_ESM.tif]

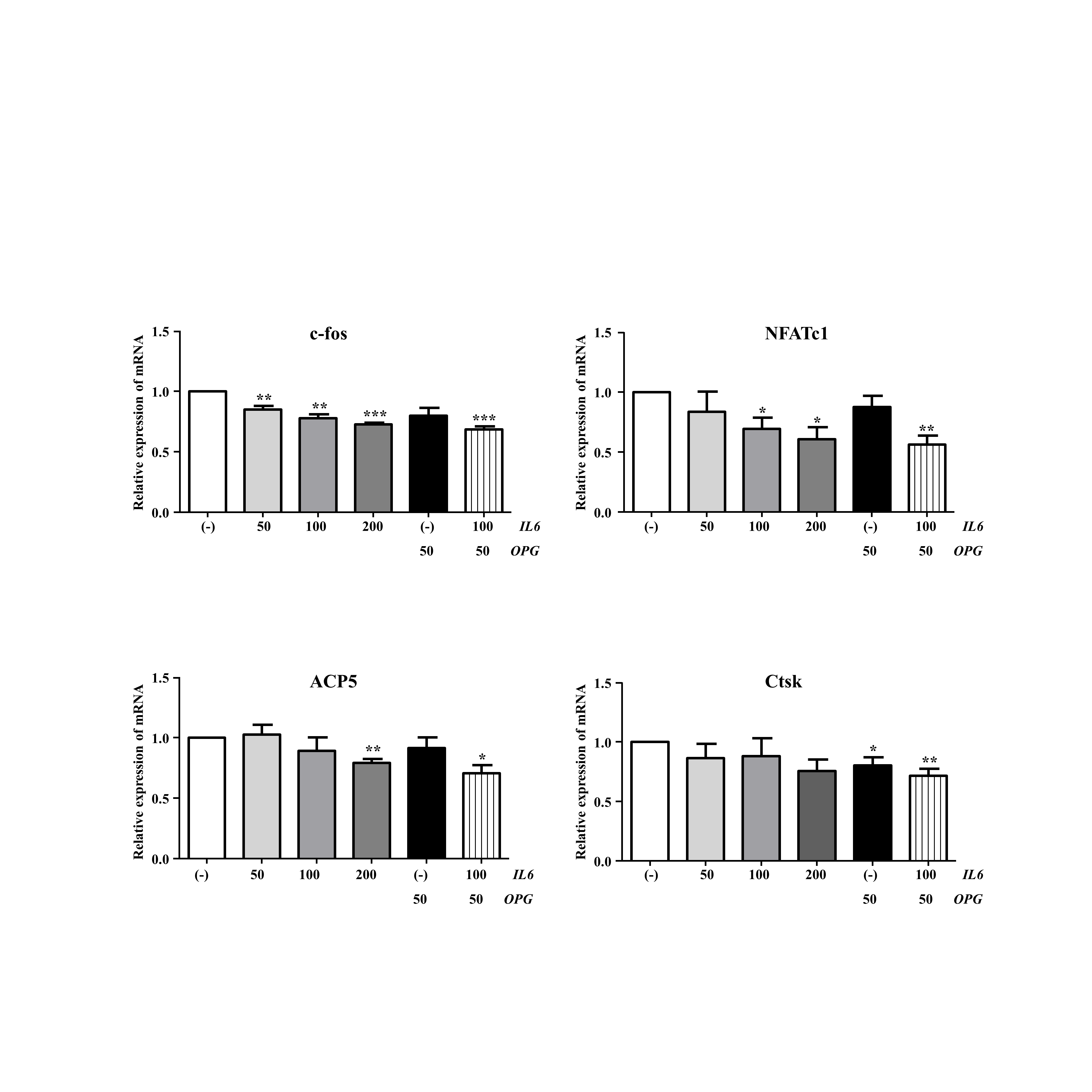

Supplement: Supplementary file 2 — Supplementary file2 (TIF 942 kb) [file 13770_2021_350_MOESM2_ESM.tif]

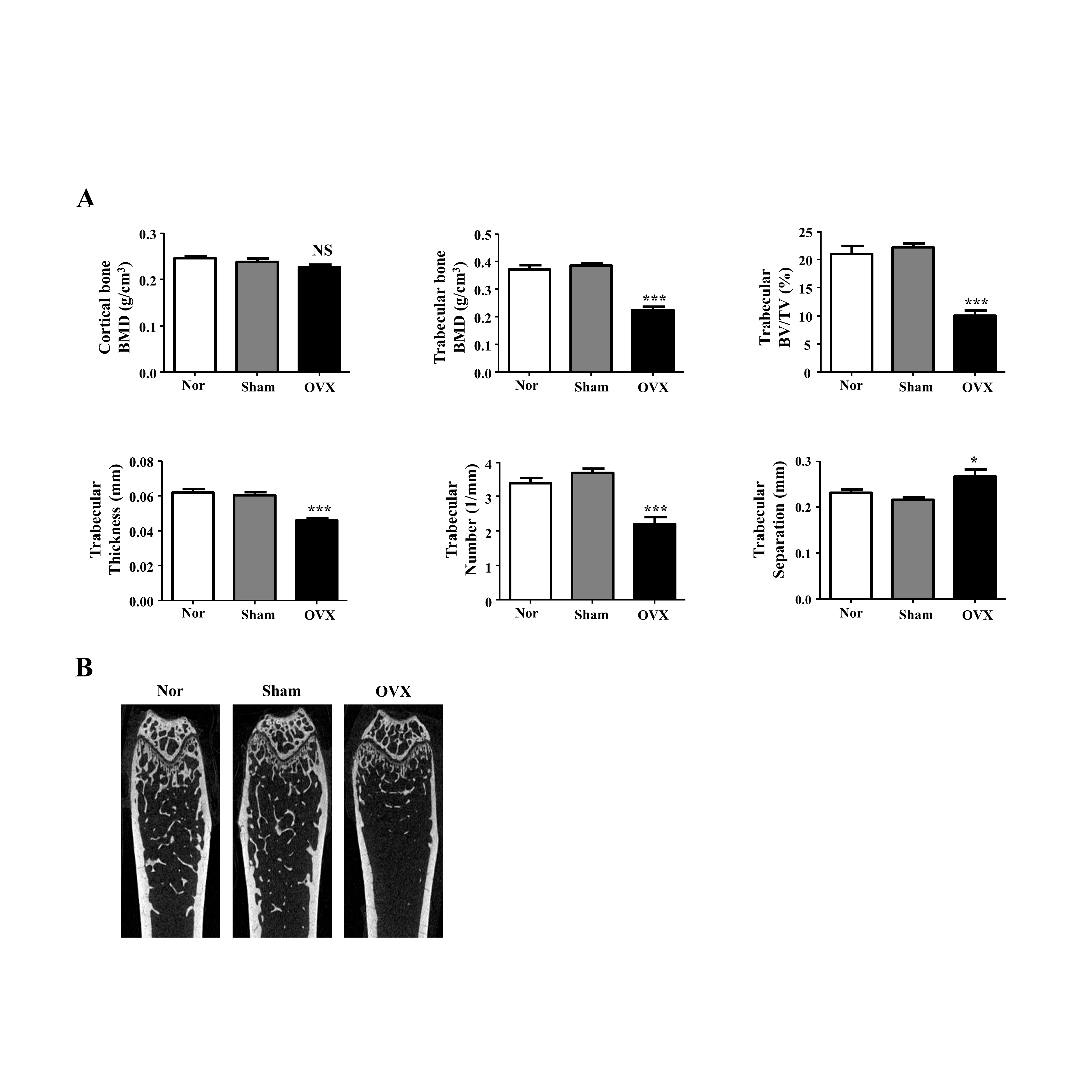

Supplement: Supplementary file 3 — Supplementary file3 (TIF 1992 kb) [file 13770_2021_350_MOESM3_ESM.tif]
